# Supplementary material for: Seasonal influence of snow conditions on Dall’s sheep productivity in Wrangell-St Elias National Park and Preserve
Source: PLoS One. 2021 Feb 9;16(2):e0244787. doi: 10.1371/journal.pone.0244787 (PMC7872280; doi:10.1371/journal.pone.0244787)
Supplement: S1 Appendix — (PDF) [file pone.0244787.s001.pdf]

Supplementary information

Table 1; Northern Wrangell-St. Elias National Park and Preserve (WRST) sheep surveys 1981 to 2017 conducted by the Alaska Department of Fish and Games (ADF&G) and the National Park Service (NPS); 41 surveys across 19 years in 9 survey units had a mean lamb-to-ewe ratio of 0.30 (SD ±0.10).

Year	Survey unit	Survey unit name	Survey agency	Date(s) of Survey	Ewes	Lambs	Lamb:Ewe	Sum
1981	4W	Nikonda Creek	ADF&G Tok	26 June, 28 June, 7 July, and 13 July	547	220	0.40	767
	4E	Cross Creek	ADF&G Tok	26 June, 28 June, 7 July, and 13 July	147	46	0.31	193
	5W	Stone Creek	ADF&G Tok	21-22 June	474	153	0.32	627
	5E	Mt Allen	ADF&G Tok	21-22 June	224	81	0.36	305
	7W	Chisana	ADF&G Tok	26-27 June	470	208	0.44	678
	10	Mount Drum	ADF&G Glennallen	Unknown	107	59	0.55	166
1984	5W	Stone Creek	ADF&G Tok	16-17 July	278	74	0.27	352
	5E	Mt Allen	ADF&G Tok	16-17 July	231	63	0.27	294
	7W	Chisana	ADF&G Tok	16-17 July	392	173	0.44	565
1987	1	Mentasta	ADF&G Tok	17 and 31 July, and 1 August	771	287	0.37	1058
1993	3	Jacksina	NPS	23 July	1628	144	0.09	1772
1997	1	Mentasta	ADF&G Tok	15, 17, 27 July	692	196	0.28	888
1998	7W	Chisana	NPS	4 August	373	118	0.32	491
1999	7W	Chisana	NPS	8 July	336	127	0.38	463
2001	10	Mount Drum	ADF&G Glennallen	12 July	65	13	0.20	78
	5E	Mt Allen	ADF&G Tok	22 July	215	27	0.13	242
	5W	Stone Creek	ADF&G Tok	22 July	301	63	0.21	364
2002	1	Mentasta	NPS	2 August	575	123	0.21	698
	2	Mount Sanford	NPS	2 August	105	38	0.36	143
	10	Mount Drum	ADF&G Glennallen	31 July	53	13	0.25	66
	7W	Chisana	ADF&G Tok	21 July	270	76	0.28	346
2005	7W	Chisana	NPS	27 July	260	63	0.24	323
2006	4E	Cross Creek	ADF&G Tok	2 August	65	25	0.38	90
	4W	Nikonda Creek	ADF&G Tok	2-3 August	315	136	0.43	451
2007	5W	Stone Creek	ADF&G Tok	28 June and 15 July	209	66	0.32	275
2011	4E	Cross Creek	ADF&G Tok	25 June	72	29	0.40	101
	5E	Mt Allen	ADF&G Tok	9 July	142	35	0.25	177
	7W	Chisana	ADF&G Tok	24-25 June	235	59	0.25	294
2012	1	Mentasta	NPS	30-31 July	664	167	0.25	831
	3	Jacksina	NPS	20, 28, and 30 July	1064	202	0.19	1266
2013	2	Mount Sanford	NPS	2 August	79	12	0.15	91
	4W	Nikonda Creek	NPS	31 July	308	67	0.22	375
2014	1	Mentasta	ADF&G Tok	22 July	360	81	0.23	441
	2	Mount Sanford	NPS	21 July	102	19	0.19	121
	5W	Stone Creek	NPS	17 July	162	41	0.25	203
	7W	Chisana	ADF&G Tok	10 July	252	95	0.38	347
2015	4E	Cross Creek	ADF&G Tok	3 August	55	22	0.40	77
	4W	Nikonda Creek	ADF&G Tok	3 August	359	120	0.33	479
2016	1	Mentasta	ADF&G Tok	15 and 27 July	555	123	0.22	678
	5E	Mt Allen	NPS	29 July	183	54	0.30	237
2017	3	Jacksina	ADF&G Tok and Glennallen	22-23 July	1386	490	0.35	1876

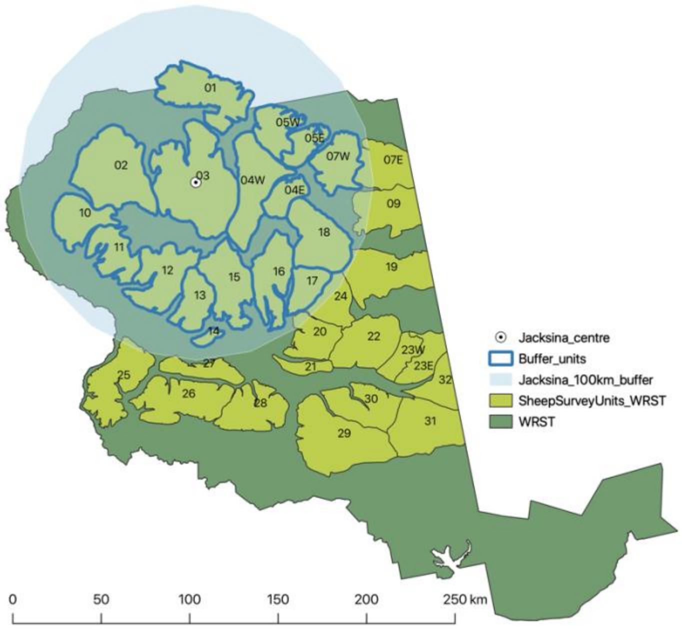

Fig 1; Map of Wrangell-St. Elias National Park and Preserve (WRST; dark green), including; all WRST Survey Units (SheepSurveyUnits_WRST; light green, numbered); 100 km radius buffer (Jacksina_100km_buffer; transparent light blue) from the centre of the Jacksina Survey Unit (Survey Unit 03; Jacksina_centre); Survey Units with centres within the 100 km radius buffer (Buffer_units, dark blue outline). GIS data for sheep survey units and WRST park boundary were sourced from [1,2] respectively.

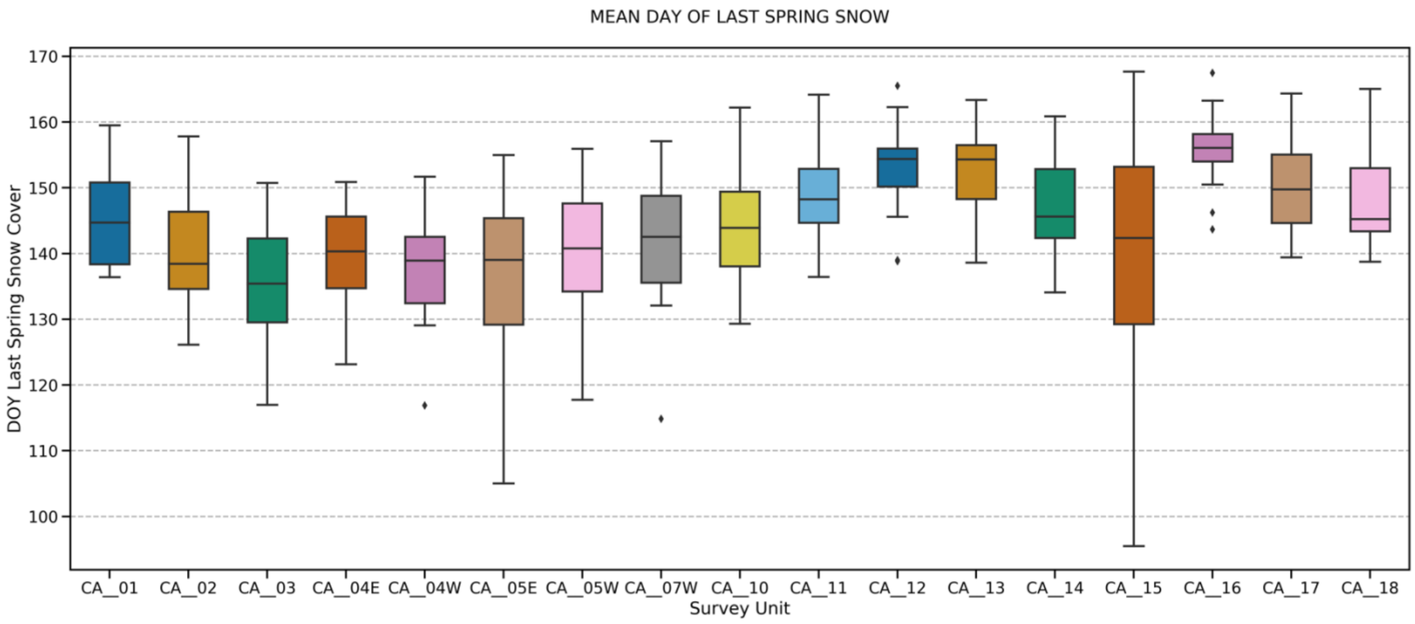

Fig 2; Box plot of the mean day of last spring snow cover in Dall's sheep habitat by Survey Unit from 2000 to 2016. Date reported as Day of Year (DOY).

To calculate the mean last day of spring snow (SDD) for a given year by Survey Unit we used Verbyla's [3] MODIS derived product for the years 2000 to 2016, please see the data reference for further details. To constrain the SDD to areas of Dall's sheep habitat we resampled the National Land Cover Database 2011 (NLCD) product [4] to the same projection and resolution as the SDD product, calculating the majority land cover in each of the 500 m SDD pixels from the higher resolution 30 m NLCD product. We then clipped the yearly raster maps of SDD and the resampled NLCD product by each individual Survey Unit included in the 100 km buffer from the centre of the Jacksina Survey Unit (see Fig S1). Finally, we found the yearly mean SDD for each Survey Unit by finding all pixels that were either Shrub/Scrub or Barren Land in the clipped NLCD raster of each Survey Unit. These two land cover types are known to be preferentially selected by Dall's sheep [5]. The corresponding pixels to these land covers were then used to find the mean SDD of each year for each Survey Unit.

Table 2; NLCD 2001 to NLCD 2011 Land Cover Change across Survey Units 1, 2, 3, 4E, 4W, 5E, 5W, 7W and 10 in the Northern Wrangell St Elias National Park and Preserve (WRST)

Code	Area km-2	Area %	Changed?	Classification
23	6.00	0.069	No	Open Water to Open Water
29	0.32	0.004	Yes	Open Water to Barren Land
44	0.02	0.000	Yes	Perennial Ice/Snow to Open Water
45	1210.31	13.947	No	Perennial Ice/Snow to Perennial Ice/Snow
50	1.51	0.017	Yes	Perennial Ice/Snow to Barren Land
149	0.39	0.005	Yes	Barren Land to Open Water
150	25.18	0.290	Yes	Barren Land to Perennial Ice/Snow
155	3592.57	41.400	No	Barren Land to Barren Land
177	15.58	0.180	No	Deciduous Forest to Deciduous Forest
191	0.02	0.000	Yes	Evergreen Forest to Open Water
199	114.50	1.319	No	Evergreen Forest to Evergreen Forest
221	25.64	0.296	No	Mixed Forest to Mixed Forest
233	0.00	0.000	Yes	Dwarf Scrub to Open Water
234	0.01	0.000	Yes	Dwarf Scrub to Perennial Ice/Snow
243	1233.50	14.214	No	Dwarf Scrub to Dwarf Scrub
254	0.01	0.000	Yes	Shrub/Scrub to Open Water
255	0.01	0.000	Yes	Shrub/Scrub to Perennial Ice/Snow
262	0.02	0.000	Yes	Shrub/Scrub to Evergreen Forest
265	2400.22	27.659	No	Shrub/Scrub to Shrub/Scrub
275	0.00	0.000	Yes	Grassland/Herbaceous to Open Water
287	7.74	0.089	No	Grassland/Herbaceous to Grassland/Herbaceous
309	2.41	0.028	No	Sedge/Herbaceous to Sedge Herbaceous
401	0.01	0.000	Yes	Woody Wetlands to Open Water
419	41.70	0.481	No	Woody Wetlands to Wood Wetlands
441	0.08	0.001	No	Herbaceous Wetlands to Herbaceous Wetlands
Total Area	8677.75	100.00		
Area unchanged	8650.2582	99.68		
Area changed	27.4932	0.32		

For the selected Survey Units (see *Survey Unit Selection* in manuscript) we calculated the amount and type of land cover change using the NLCD 2011 Land Cover Alaska 2001 to 2011 From To Change Index [6]. To do this we first clipped the NLCD Land Cover Alaska 2001 to 2011 product to all the 9 selected units. Using the clipped layer we then ran the Raster Layer Unique Values Report in QGIS [7] to find the area of each type of change and simply calculated the area changed or unchanged using the product's change classification scheme (Table S1).

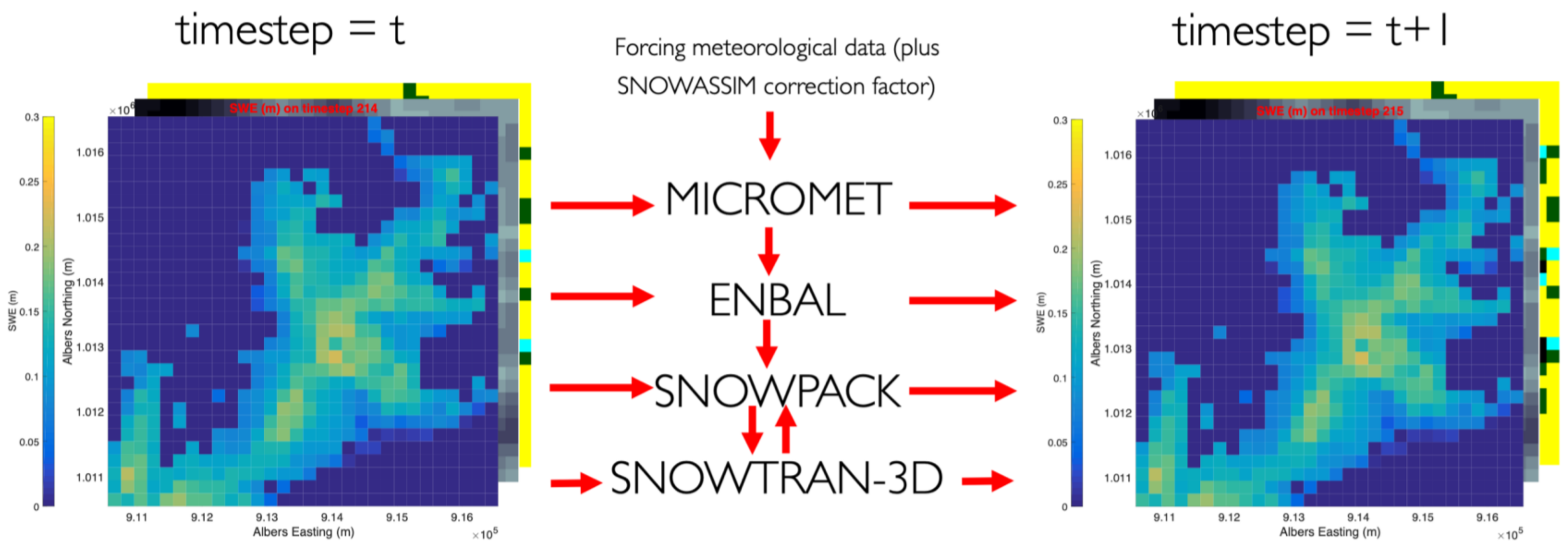

Figure 3; workflow diagram of SnowModel showing the interactions between each sub-model, meteorological forcing data (corrected by DataAssim), static layers (vegetation and elevation set furthest back and behind example spatially distributed SWE output respectively) and the previous timestep's snow condition. Full descriptions can be found for each submodel in the following references; MicroMet [8], EnBal [9], SnowPack [10], SnowTran-3D [11], and SnowAssim [12]

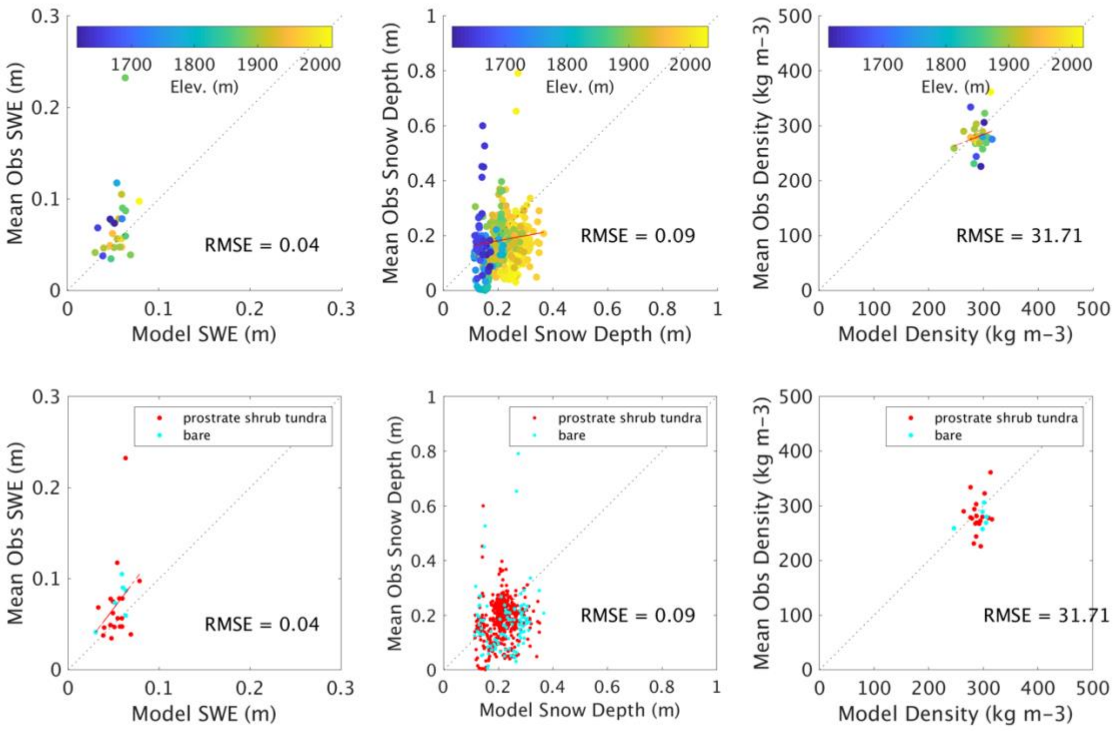

Fig 4; Best calibration (ro_adj = 6.0; wspd_increase = 2.5) model vs observed SWE, Snow Depth and Snow Density by elevation and land cover class

After an initial sensitivity analysis, our calibration involved 72 SnowModel simulations from 1st September 2016 to 1st April 2017 with the density adjustment factor (*ro_adj*) ranging from 2.0 to 10.0 in increments of 1.0, and the wind speed scalar (*wspd_increase*) ranging from 1.5 to 5.0 in increments of 0.5.

Table 3; RMSE for SWE, depth and density for each calibration simulation. Ranking is determined by minimum RMSE and Mean ranking is used to select the best calibration.

Parameters	RMSE SWE (m)	RMSE Depth (m)	RMSE Density (kg m-3)	SWE rank	Depth rank	Density rank	Mean ranking
ro_adj06.0-wspd2.5	0.04	0.09	31.71	5	13	6	**8.00**
ro_adj07.0-wspd2.5	0.04	0.09	34.30	6	9	10	**8.33**
ro_adj08.0-wspd2.5	0.04	0.09	37.22	7	6	15	**9.33**
ro_adj04.0-wspd2.5	0.04	0.10	30.45	4	24	5	**11.00**
ro_adj09.0-wspd2.5	0.04	0.09	40.23	8	5	22	**11.67**
ro_adj10.0-wspd2.5	0.04	0.09	43.19	9	4	27	**13.33**
ro_adj03.0-wspd2.5	0.04	0.10	34.53	3	33	12	**16.00**
ro_adj15.0-wspd2.5	0.04	0.09	48.76	10	3	36	**16.33**
ro_adj20.0-wspd2.5	0.04	0.08	66.29	11	2	56	**23.00**
ro_adj09.0-wspd3.0	0.06	0.10	33.05	44	21	9	**24.67**
ro_adj08.0-wspd3.0	0.06	0.10	34.35	43	20	11	**24.67**
ro_adj10.0-wspd3.0	0.06	0.10	32.34	45	22	8	**25.00**
ro_adj07.0-wspd3.0	0.06	0.10	36.39	42	19	14	**25.00**
ro_adj25.0-wspd2.5	0.04	0.08	69.72	12	1	62	**25.00**
ro_adj15.0-wspd3.0	0.06	0.10	32.24	46	26	7	**26.33**
ro_adj06.0-wspd3.0	0.06	0.10	39.37	41	18	21	**26.67**
ro_adj02.0-wspd2.5	0.04	0.11	44.75	1	50	30	**27.00**
ro_adj05.0-wspd2.5	0.04	0.11	44.75	2	51	31	**28.00**
ro_adj04.0-wspd3.0	0.06	0.09	49.45	40	14	37	**30.33**
ro_adj03.0-wspd3.0	0.06	0.09	57.70	39	10	45	**31.33**
ro_adj20.0-wspd3.0	0.06	0.10	38.88	48	29	19	**32.00**

ro_adj25.0-wspd3.0	0.06	0.10	41.00	47	30	23	**33.33**
ro_adj02.0-wspd3.0	0.06	0.09	70.12	37	11	63	**37.00**
ro_adj05.0-wspd3.0	0.06	0.09	70.12	38	12	64	**38.00**
ro_adj02.0-wspd2.0	0.04	0.18	29.07	23	91	1	**38.33**
ro_adj05.0-wspd2.0	0.04	0.18	29.07	24	92	2	**39.33**
ro_adj06.0-wspd3.5	0.06	0.10	52.00	53	28	39	**40.00**
ro_adj07.0-wspd3.5	0.06	0.10	48.34	54	34	33	**40.33**
ro_adj03.0-wspd2.0	0.04	0.16	34.83	22	87	13	**40.67**
ro_adj15.0-wspd3.5	0.06	0.11	38.87	60	44	18	**40.67**
ro_adj09.0-wspd3.5	0.06	0.11	43.16	58	38	26	**40.67**
ro_adj10.0-wspd3.5	0.06	0.11	41.35	59	41	24	**41.33**
ro_adj20.0-wspd3.5	0.06	0.12	37.45	56	53	16	**41.67**
ro_adj08.0-wspd3.5	0.06	0.11	45.45	57	36	32	**41.67**
ro_adj04.0-wspd3.5	0.06	0.10	62.66	52	23	50	**41.67**
ro_adj25.0-wspd3.5	0.06	0.12	38.18	55	55	17	**42.33**
ro_adj04.0-wspd2.0	0.04	0.15	42.80	21	83	25	**43.00**
ro_adj02.0-wspd1.5	0.05	0.23	29.97	35	95	3	**44.33**
ro_adj03.0-wspd3.5	0.06	0.09	70.79	51	17	65	**44.33**
ro_adj02.0-wspd3.5	0.06	0.09	82.56	49	7	79	**45.00**
ro_adj05.0-wspd1.5	0.05	0.23	29.97	36	96	4	**45.33**
ro_adj05.0-wspd3.5	0.06	0.09	82.56	50	8	80	**46.00**
ro_adj20.0-wspd2.0	0.04	0.11	104.03	14	40	91	**48.33**
ro_adj25.0-wspd2.0	0.04	0.11	107.94	16	35	94	**48.33**
ro_adj06.0-wspd2.0	0.04	0.14	56.65	20	82	44	**48.67**
ro_adj07.0-wspd2.0	0.04	0.13	62.37	19	79	48	**48.67**
ro_adj03.0-wspd1.5	0.05	0.21	39.13	34	94	20	**49.33**
ro_adj08.0-wspd2.0	0.04	0.13	67.44	18	76	58	**50.67**
ro_adj06.0-wspd4.0	0.06	0.11	62.44	65	39	49	**51.00**
ro_adj10.0-wspd2.0	0.04	0.12	76.12	15	65	73	**51.00**

ro_adj15.0-wspd2.0	0.04	0.12	83.35	13	59	81	**51.00**
ro_adj07.0-wspd4.0	0.06	0.11	58.89	66	42	46	**51.33**
ro_adj09.0-wspd2.0	0.04	0.13	72.00	17	71	66	**51.33**
ro_adj25.0-wspd4.0	0.06	0.12	44.01	67	61	28	**52.00**
ro_adj09.0-wspd4.0	0.06	0.11	53.60	70	46	40	**52.00**
ro_adj20.0-wspd4.0	0.06	0.12	44.12	68	60	29	**52.33**
ro_adj10.0-wspd4.0	0.06	0.11	51.63	71	48	38	**52.33**
ro_adj08.0-wspd4.0	0.06	0.11	55.99	69	45	43	**52.33**
ro_adj15.0-wspd4.0	0.06	0.11	48.64	72	52	35	**53.00**
ro_adj04.0-wspd4.0	0.06	0.10	72.58	64	27	68	**53.00**
ro_adj04.0-wspd1.5	0.05	0.19	48.48	33	93	34	**53.33**
ro_adj02.0-wspd4.0	0.06	0.09	91.20	61	15	85	**53.67**
ro_adj03.0-wspd4.0	0.06	0.10	80.19	63	25	76	**54.67**
ro_adj05.0-wspd4.0	0.06	0.09	91.20	62	16	86	**54.67**
ro_adj06.0-wspd1.5	0.05	0.18	63.48	32	90	52	**58.00**
ro_adj07.0-wspd1.5	0.05	0.17	69.49	31	89	61	**60.33**
ro_adj25.0-wspd4.5	0.06	0.12	54.43	77	64	41	**60.67**
ro_adj20.0-wspd4.5	0.06	0.12	54.82	78	62	42	**60.67**
ro_adj15.0-wspd4.5	0.06	0.12	59.94	83	58	47	**62.67**
ro_adj07.0-wspd4.5	0.06	0.11	69.41	80	49	59	**62.67**
ro_adj08.0-wspd1.5	0.05	0.17	74.79	30	88	71	**63.00**
ro_adj09.0-wspd1.5	0.05	0.16	79.52	28	86	75	**63.00**

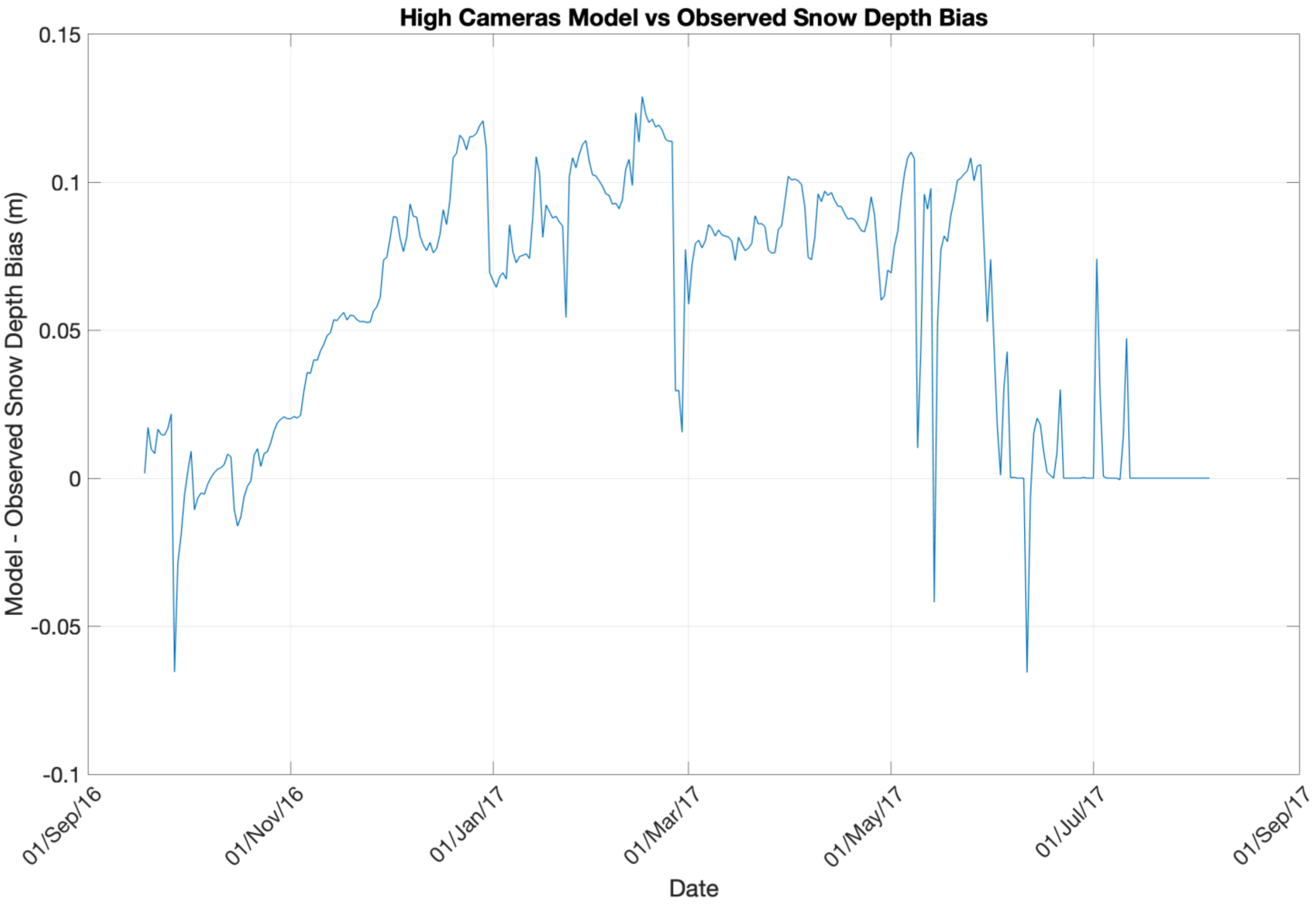

Figure 5; Modelled vs Observed snow depth bias through time from the best calibration (ro_adj = 6.0; wspd_increase = 2.5) to observations of snow depth from cameras located at high elevations above shrubline. Mean bias is 0.06 cm.

Table 4; Summary statistics of model-derived snow and climate covariates by season within the Jacksina sheep survey unit within Wrangell-St. Elias National Park and Preserve, Alaska.

Variable	Season	N	Mean	SD	CV	Min	25%	75%	Max
Snow Depth (m)	Fall	37	0.25	0.06	22.21	0.15	0.21	0.28	0.40
	Winter	37	0.36	0.05	15.10	0.25	0.32	0.40	0.46
	Spring	37	0.37	0.06	15.45	0.23	0.33	0.41	0.47
Snow Density (kg m-3)	Fall	37	251.36	20.25	8.06	213.17	233.82	267.16	299.64
	Winter	37	283.35	16.92	5.97	247.39	272.26	294.15	316.21
	Spring	37	323.26	14.13	4.37	299.70	314.31	329.65	350.99
Forageable Area (%)	Fall	37	77.46	7.63	9.85	53.62	73.51	82.42	93.13
	Winter	37	61.59	9.52	15.46	40.70	54.57	66.58	80.99
	Spring	37	49.86	7.50	15.05	34.53	46.48	55.09	65.82
Snowfall (m)	Fall	37	0.16	0.03	21.47	0.08	0.13	0.18	0.22
	Winter	37	0.11	0.02	21.87	0.06	0.09	0.12	0.16
	Spring	37	0.10	0.02	17.32	0.07	0.09	0.11	0.14
Air Temperature (ºC)	Fall	37	-8.40	1.36	-16.14	-11.71	-9.26	-7.51	-5.05
	Winter	37	-15.27	1.92	-12.59	-20.42	-16.07	-14.01	-11.75
	Spring	37	-8.75	1.52	-17.36	-11.10	-10.04	-7.89	-5.16

Table 5; Table of single predictor models and null model showing; p-values (ANOVA P-value) for an ANOVA test between the model fitted with and without a random effect (Survey Unit) and using Restricted Maximum Likelihood; the second-order Akaike Information Criterion (AICc) of the single predictor model fitted without a random effect and by Ordinary Least Squares. Models are ranked by AICc and all predictors with an AICc greater than the Null model are taken forward into multiple predictor models (see Table 4 below)

**Variable**	**ANOVA P-value**	**AICc**
Fall Snow Depth (m)	0.64	-89.35
Spring Snow Depth (m)	0.86	-87.23
Winter Snow Depth (m)	0.77	-86.56
Fall Air Temperature (ºC)	1.00	-80.76
Spring Forageable Area (%)	0.60	-77.62
Fall Forageable Area (%)	0.22	-77.20
Fall Snowfall (m)	0.22	-76.33
Winter Snowfall (m)	0.62	-75.13
Spring Snow Density (kg m-3)	0.66	-74.99
Fall Snow Density (kg m-3)	0.26	-74.49
Spring Snowfall (m)	0.29	-74.06
Null	0.43	-73.47
Winter Forageable Area (%)	0.31	-72.01
Previous Year Air Temperature (ºC)	0.45	-71.48
Winter Snow Density (kg m-3)	0.34	-70.96
Spring Air Temperature (ºC)	0.45	-70.51
Winter Air Temperature (ºC)	0.47	-70.47
Previous Summer Snow Depth (m)	0.46	-68.98
Previous Year Snow Density (kg m-3)	0.21	-68.46
Previous Summer Snowfall (m)	0.36	-67.99
Previous Year Snow Depth (m)	0.45	-67.80
Previous Year Forageable Area (%)	0.30	-67.61
Previous Summer Forageable Area (%)	0.35	-67.57
Previous Summer Air Temperature (ºC)	0.36	-67.40
Previous Year Snowfall (m)	0.32	-67.28
Previous Summer Snow Density (kg m-3)	1.00	-17.58

*Table 6; Complete table of multi-predictor models and Null model ranked by their second-order Akaike Information Criterion (AICc). Standard error (SE) shown in brackets for both the intercept and estimate of each predictor in each model. 1st Predictor indicates the 1st snow and climate covariable listed in the Model column. ** indicates significance at a Bonferroni corrected alpha level of 0.00125 (alpha / total models); * indicates significance at alpha = 0.05. P-values were computed in R by the Wald test method via use of the 'summary' core package [13].*

Model	Intercept (SE)	1st Predictor Estimate (SE)	Fall Air Temperature (SE)	Fall Snowfall (SE)	K	Delta AICc	AICc weight	R-Sq.	Adjusted R-Sq.
Fall Snow Depth + Fall Air Temperature	0.690 (0.094)**	-0.738 (0.193)**	0.027 (0.012)*	–	3	0	0.188	0.439	0.41
Winter Snow Depth + Fall Air Temperature + Fall Snowfall	0.900 (0.112)**	-0.599 (0.214)*	0.032 (0.012)*	-0.818 (0.398)*	4	0.134	0.176	0.472	0.429
Spring Snow Depth + Fall Air Temperature + Fall Snowfall	0.851 (0.111)**	-0.522 (0.192)*	0.027 (0.013)*	-0.940 (0.388)*	4	0.523	0.145	0.467	0.424
Fall Snow Depth + Fall Air Temperature + Fall Snowfall	0.780 (0.114)**	-0.593 (0.219)*	0.030 (0.012)*	-0.593 (0.435)	4	0.592	0.14	0.466	0.423
Winter Snow Depth + Fall Air Temperature	0.792 (0.103)**	-0.738 (0.211)**	0.029 (0.012)*	–	3	1.963	0.071	0.412	0.381
Fall Snow Depth	0.511 (0.046)**	-0.895 (0.187)**	–	–	2	2.328	0.059	0.37	0.354
Spring Snow Depth + Fall Snowfall	0.689 (0.081)**	-0.720 (0.173)**	–	-0.848 (0.402)*	3	2.374	0.057	0.406	0.375
Spring Snow Depth + Fall Air Temperature	0.706 (0.099)**	-0.623 (0.199)*	0.023 (0.014)	–	3	3.949	0.026	0.383	0.35
Fall Snow Depth + Fall Snowfall	0.552 (0.068)**	-0.818 (0.211)**	–	-0.367 (0.452)	3	4.084	0.024	0.381	0.348
Spring Snow Depth	0.577 (0.064)**	-0.788 (0.177)**	–	–	2	4.45	0.02	0.336	0.319
Winter Snow Depth + Fall Snowfall	0.689 (0.086)**	-0.807 (0.215)**	–	-0.687 (0.426)	3	4.87	0.017	0.369	0.335
Winter Snow Depth	0.614 (0.074)**	-0.909 (0.210)**	–	–	2	5.121	0.015	0.326	0.308
Fall Air Temperature + Fall Snowfall	0.847 (0.120)**	0.045 (0.012)**	–	-1.170 (0.410)*	3	5.411	0.013	0.36	0.327
Fall Forageable Area + Fall Air Temperature	0.314 (0.165)	0.004 (0.002)*	0.041 (0.012)*		3	6.592	0.007	0.342	0.307
Spring Snowfall + Fall Air Temperature + Fall Snowfall	0.878 (0.123)**	-0.696 (0.622)	0.040 (0.013)*	-1.181 (0.409)*	4	6.649	0.007	0.381	0.331
Fall Forageable Area + Fall Air Temperature + Fall Snowfall	0.609 (0.257)*	0.002 (0.002)	0.043 (0.012)*	-0.802 (0.540)	4	6.817	0.006	0.379	0.328
Winter Snowfall + Fall Air Temperature + Fall Snowfall	0.881 (0.124)**	-0.712 (0.690)	0.042 (0.013)*	-1.060 (0.423)*	4	6.851	0.006	0.378	0.328
Spring Forageable Area + Fall Air Temperature + Fall Snowfall	0.699 (0.208)*	0.002 (0.002)	0.041 (0.013)*	-0.983 (0.463)*	4	7.169	0.005	0.373	0.323
Fall Snow Density + Fall Air Temperature + Fall Snowfall	0.951 (0.180)**	-0.001 (0.001)	0.044 (0.012)**	-0.959 (0.494)	4	7.353	0.005	0.371	0.32
Spring Snow Density + Fall Air Temperature + Fall Snowfall	1.013 (0.314)*	-0.001 (0.001)	0.043 (0.013)*	-1.072 (0.448)*	4	7.653	0.004	0.366	0.315
Fall Snow Density + Fall Air Temperature	0.986 (0.186)**	-0.001 (0.001)*	0.043 (0.013)*	–	3	8.732	0.002	0.306	0.27
Spring Forageable Area + Fall Air Temperature	0.407 (0.163)*	0.004 (0.002)	0.036 (0.013)*	–	3	9.274	0.002	0.297	0.26
Winter Snowfall + Fall Air Temperature	0.740 (0.118)**	-1.150 (0.712)	0.039 (0.013)*	–	3	10.658	0.001	0.273	0.235
Fall Air Temperature	0.657 (0.108)**	0.044 (0.013)*	–	–	2	10.917	0.001	0.223	0.203
Spring Snow Density + Fall Air Temperature	1.130 (0.328)*	-0.002 (0.001)	0.039 (0.014)*	–	3	10.944	0.001	0.268	0.229
Spring Snowfall + Fall Air Temperature	0.684 (0.112)**	-0.656 (0.680)	0.040 (0.014)*	–	3	12.386	0	0.242	0.202
Spring Snowfall + Fall Snowfall	0.622 (0.099)**	-1.357 (0.645)*	–	-1.181 (0.451)*	3	13.289	0	0.225	0.184
Spring Forageable Area	0.037 (0.096)	0.005 (0.002)*	–		2	14.054	0	0.161	0.14
Spring Forageable Area + Fall Snowfall	0.224 (0.158)	0.004 (0.002)	–	-0.746 (0.508)	3	14.247	0	0.206	0.165
Fall Forageable Area	-0.059 (0.135)	0.005 (0.002)*	–	–	2	14.48	0	0.153	0.131
Winter Snowfall + Fall Snowfall	0.591 (0.098)**	-1.277 (0.750)	–	-0.961 (0.474)*	3	14.795	0	0.196	0.153
Fall Snowfall	0.483 (0.077)**	-1.158 (0.471)*	–	–	2	15.346	0	0.134	0.112
Spring Snow Density + Fall Snowfall	0.959 (0.352)*	-0.002 (0.001)	–	-0.902 (0.501)	3	15.79	0	0.176	0.133
Fall Forageable Area + Fall Snowfall	0.156 (0.252)	0.003 (0.002)	–	-0.619 (0.611)	3	15.848	0	0.175	0.131
Winter Snowfall	0.477 (0.084)**	-1.649 (0.756)*	–	–	2	16.546	0	0.109	0.086
Spring Snow Density	1.063 (0.357)*	-0.002 (0.001)*	–	–	2	16.687	0	0.106	0.083
Fall Snow Density + Fall Snowfall	0.619 (0.177)**	-0.001 (0.001)	–	-0.894 (0.566)	3	17.038	0	0.151	0.106
Fall Snow Density	0.657 (0.179)**	-0.001 (0.001)	–	–	2	17.186	0	0.095	0.072
Spring Snowfall	0.428 (0.070)**	-1.316 (0.691)	–	–	2	17.619	0	0.085	0.062
Null	0.290 (0.015)**	–	–	–	1	18.207	0	–	–

Table 7; Coefficients and fit statistics of the linear models testing for trends in each variable by season from 1980 to 2017

Variable	Season	Estimate	R-squared	P-value
Snow Depth (m)	Fall	-0.001	0.023	0.37
	Winter	0.000	0.009	0.58
	Spring	-0.001	0.019	0.41
Snow Density (kg m-3)	Fall	-0.322	0.030	0.31
	Winter	-0.211	0.018	0.43
	Spring	-0.208	0.025	0.35
Forageable Area (%)	Fall	0.100	0.020	0.40
	Winter	0.120	0.018	0.42
	Spring	0.150	0.047	0.20
Snowfall (m)	Fall	0.000	0.004	0.71
	Winter	0.000	0.009	0.57
	Spring	0.000	0.040	0.23
Air Temperature (°C)	Fall	0.033	0.068	0.12
	Winter	0.038	0.046	0.20
	Spring	0.002	0.000	0.93

Table 8; Coefficients and fit statistics of the linear models testing for trends in the rolling 10-year coefficient of variation of each variable by season from 1980 to 2017

Variable	Season	Estimate	R-squared	P-value
Snow Depth (m)	Fall	-0.019	0.002	0.81
	Winter	0.041	0.018	0.46
	Spring	-0.038	0.011	0.57
Snow Density (kg m-3)	Fall	0.027	0.068	0.14
	Winter	0.057	0.292	0.00
	Spring	-0.002	0.000	0.93
Forageable Area (%)	Fall	0.051	0.028	0.35
	Winter	-0.080	0.065	0.15
	Spring	-0.019	0.011	0.55
Snowfall (m)	Fall	0.050	0.012	0.54
	Winter	-0.165	0.254	0.00
	Spring	0.087	0.064	0.16
Air Temperature (°C)	Fall	0.076	0.123	0.05
	Winter	0.005	0.000	0.92
	Spring	0.002	0.000	0.96

References

[1] WRST Sheep and Goat Count Units - data.doi.gov n.d. https://data.doi.gov/dataset/wrst-sheep-and-goat-count-units (accessed May 24, 2020).

[2] National Park Boundaries - Data.gov n.d. https://catalog.data.gov/dataset/national-park-boundariesf0a4c (accessed May 24, 2020).

[3] Verbyla D. ABoVE: Last Day of Spring Snow, Alaska, USA, and Yukon Territory, Canada, 2000-2016. ORNL DAAC 2017. https://doi.org/10.3334/ORNLDAAC/1528.

[4] Homer C, Dewitz J, Yang L, Jin S, Danielson P. Completion of the 2011 National Land Cover Database for the Conterminous United States – Representing a Decade of Land Cover Change Information. PHOTOGRAMMETRIC ENGINEERING 2015:11.

[5] Mahoney PJ, Liston GE, LaPoint S, Gurarie E, Mangipane B, Wells AG, et al. Navigating snowscapes: scale-dependent responses of mountain sheep to snowpack properties. Ecological Applications 2018;0. https://doi.org/10.1002/eap.1773.

[6] U.S. Geological Survey. NLCD 2011 Land Cover Alaska 2001 to 2011 From To Change Index - National Geospatial Data Asset (NGDA) Land Use Land Cover 2015. https://www.mrlc.gov/data/nlcd-2001-2011-land-cover-change-alaska-0.

[7] QGIS Development Team. QGIS Geographic Information System. Open Source Geospatial Foundation Project; 2019.

[8] Liston GE, Elder K, Liston GE, Elder K. A Meteorological Distribution System for High-Resolution Terrestrial Modeling (MicroMet). Journal of Hydrometeorology 2006;7:217–234. https://doi.org/10.1175/JHM486.1.

[9] Liston GE. Local Advection of Momentum, Heat, and Moisture during the Melt of Patchy Snow Covers. J Appl Meteor 1995;34:1705–15. https://doi.org/10.1175/1520-0450-34.7.1705.

[10] Liston GE, Hall DK. An energy-balance model of lake-ice evolution. Journal of Glaciology 1995;41:373–82. https://doi.org/10.3189/S0022143000016245.

[11] Liston GE, Haehnel RB, Sturm M, Hiemstra CA, Berezovskaya S, Tabler RD. Simulating complex snow distributions in windy environments using SnowTran-3D. Journal of Glaciology 2007;53:241–56. https://doi.org/10.3189/172756507782202865.

[12] Liston GE, Hiemstra CA. A Simple Data Assimilation System for Complex Snow Distributions (SnowAssim). J Hydrometeor 2008;9:989–1004. https://doi.org/10.1175/2008JHM871.1.

[13]R Core Team. R: A Language and Environment for Statistical Computing. Vienna, Austria: R Foundation for Statistical Computing; 2019.
