# Supplementary figures and images for: Seasonal influence of snow conditions on Dall’s sheep productivity in Wrangell-St Elias National Park and Preserve
Source: PLoS One. 2021 Feb 9;16(2):e0244787. doi: 10.1371/journal.pone.0244787 (PMC7872280; doi:10.1371/journal.pone.0244787)

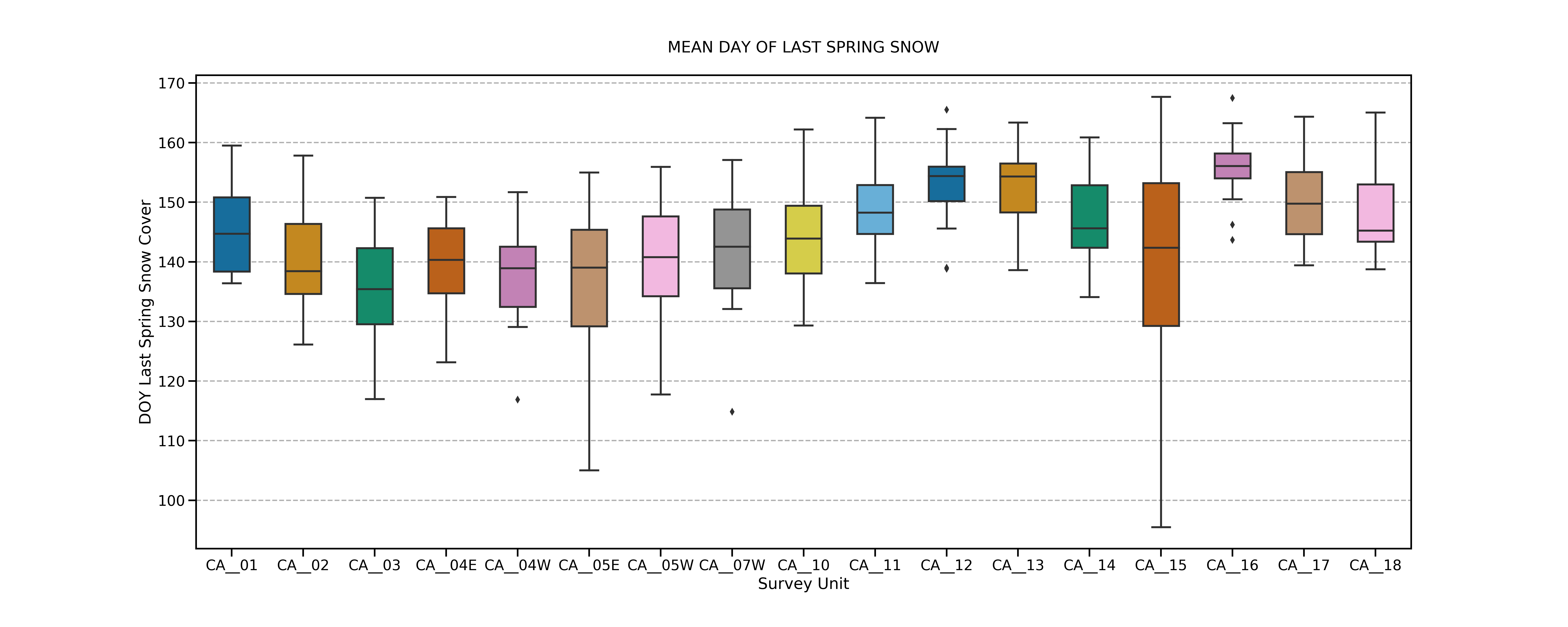

Supplement: S6 File — (ZIP) [file pone.0244787.s015.zip › S14_create_fig_A2/SDD_by_survey_unit.png]

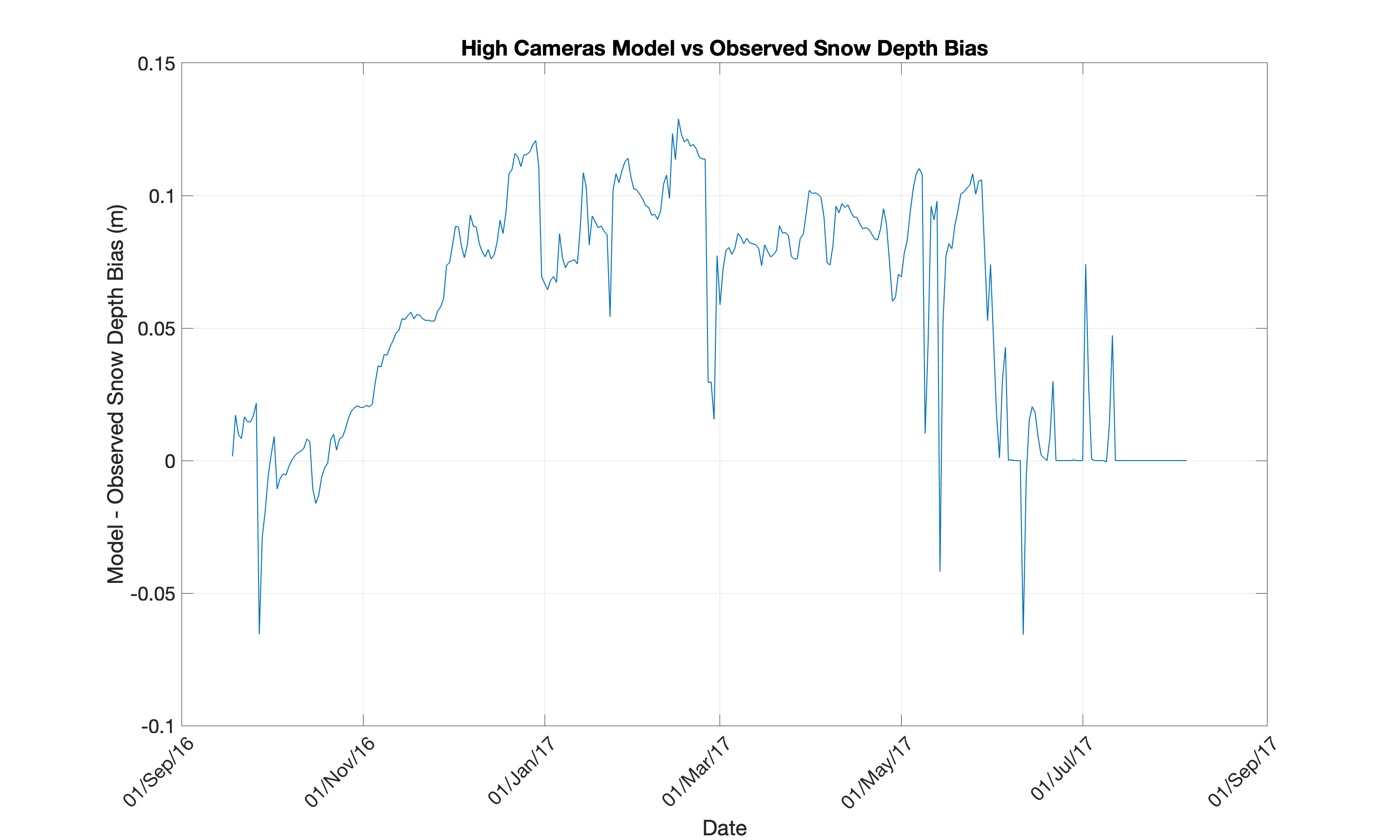

Supplement: S8 File — (ZIP) [file pone.0244787.s017.zip › model_v_obs_bias.png]
