# Supplementary material for: Seasonal influence of snow conditions on Dall’s sheep productivity in Wrangell-St Elias National Park and Preserve
Source: PLoS One. 2021 Feb 9;16(2):e0244787. doi: 10.1371/journal.pone.0244787 (PMC7872280; doi:10.1371/journal.pone.0244787)
Supplement: Attachment — Submitted filename: Larue_Review_11_03_2020.pdf [file pone.0244787.s020.pdf]

Comments to the Author

This manuscript explores how different snow properties affect the population dynamics of a northern alpine ungulate. More precisely, it explores the effects of snow depth, snow density, forageable area and snowfall on lamb to ewe ratios in Dall sheep. The authors use a spatially-explicit snow evolution model to determine snow cover properties. Subsequently, they evaluate the correlation between these properties and lamb to ewe ratios obtained from periodic summer aerial surveys which spanned over 37 years.

Overall, the manuscript is well structured and written. However, I think it could be slightly shortened by cutting certain sentences and avoiding excessive repetition of the same information in different sections. The analytical approach is elegant and convincing. However, I have never used nor read about spatially-explicit snow evolution models for my own research and I am not an expert on this specific matter. The research question isn't so novel, but the methods to address it are. The results from this manuscript are hence novel and will be, in my opinion, of interest for management and conservation of ungulates and other species living in snowy environments. I enjoyed reviewing this manuscript and mostly have minor comments except for the advised use of sequential regression to confront multicollinearity and the over-interpretation of the AICc model selection. Fitting models with multiple snow properties and their interactions as covariates could greatly improve explanatory power and give a more accurate picture of the total effect of snow properties on Dall's sheep productivity. I also think this manuscript could be improved and broadened be making it less "Dall sheep centric". The application of SnowModel could also be interesting for studies of many plant and animal species living in snowy environments.

Introduction

The introduction could be slightly shortened by using less examples, more concise writing and less repetition. For example, at lines 116-117, the method used to determine the lamb to ewe ratios (aerial surveys) doesn't need to be mentioned here and is simply a repetition of the what should remain in the methods.

Line 42: I don't think the reference to global climate systems is relevant to this manuscript.

Lines 55-61: Despite mentioning muskoxen, there is no citation pertaining to the species but there are 2 on deer. I think a citation on muskoxen is warranted.

Lines 86-88: I understand the idea here, but are there examples of studies where point-locations were non-representative? Though it is only one example, at my study site, which is also in an

alpine environment, temperature is highly correlated (>0.85) to that of the nearest meteorological station in a valley-bottom ~15km away.

Lines 98-101: "These fluctuations are thought to be largely governed by variations in the production and survival of lambs" – The authors should also cite relevant articles that have highlighted the greater importance of juvenile survival and recruitment in ungulate population dynamics such as Gaillard et al. 1998 (Population dynamics of large herbivores: variable recruitment with constant adult survival; https://doi.org/10.1016/S0169-5347(97)01237-8) or Gaillard et al. 2000 (Temporal Variation in Fitness Components and Population Dynamics of Large Herbivores; https://doi.org/10.1146/annurev.ecolsys.31.1.367).

Lines 108-109: Citation on forage accessibility? References 79 or 80 from this manuscript?

Lines 122-128: Very interesting contrasting hypotheses, but I don't think the word predict should be used here. This study doesn't exactly assess predictive power/accuracy using recognised methods such as cross-validation. In my opinion, a rephrasing like "…snow conditions established in the fall months should explain more variance in summer lamb-to-ewe ratios." would be more appropriate.

Methods

Line 142-143: Are average temperatures essential?

Lines 143-147: The description of lower elevation vegetation is not necessary and lengthy. It is indicated at line 151 that sheep range starts at around 1400 m. Also, it is later indicated that only pixels above 1200 m were selected at line 319.

Lines 194-195: Aerial surveys do not offer perfect detection. This could be especially true in Dall sheep which have a fission-fusion aggregation dynamic in which group size and composition can drastically change over the year. Potential biases of aerial surveys and the minimum count method have already been studied in Dall sheep, e.g. Udevitz et al. 2010 (Evaluation of Aerial Survey Methods for Dall's Sheep; https://doi.org/10.2193/0091-7648(2006)34[732:EOASMF]2.0.CO;2) and Schmidt et al. 2012 (Using distance sampling and hierarchical models to improve estimates of Dall's sheep abundance; https://doi.org/10.1002/jwmg.216). Schmidt et al (2012) even highlight a bias in lamb abundance estimates which could be a serious issue in this manuscript. A sentence relating to the potential biases of these methods should be added.

Lines 206-209: I think this table should be in an appendix. It is lengthy and a sentence or two with descriptive statistics in the text (e.g. min and max date of flight, min and max number of individuals reported…) could replace it.

Lines 210-346: I am not familiar with the specifics of SnowModel and its calibration, nor am I an expert on snow properties. I thus cannot comment these specific aspects.

Line 307: Is this not Root Mean Squared Error?

Lines 318-333: This section could be condensed into only 3-4 short sentences by greatly cutting down on the descriptions of the cited studies (e.g. only including the citation is enough for the choice of altitudinal range).

Line 343: How high is half-chest height?

Lines 345-346: I would add "…Dall's sheep productivity…" to avoid confusion with plant productivity because this part refers to forage.

Lines 349-350: The developers of the R software should be cited. Use citation() in R to obtain the appropriate citation (https://astrostatistics.psu.edu/su07/R/library/utils/html/citation.html)

Lines 350-352: The authors should consider including the date of the survey in their models given the relatively wide timespan in survey date (26 June to 4 August) and potential lamb summer mortality. I doubt this will have a large effect, but it is worth evaluating.

Lines 354-355: Rather than including only one snow property variable per model, all four variables, a combination of variables and/or a combination of variables and their interactions should be tested using a sequential regression method. This exercise could be very informative if, as I expect (given lines 404-407), the full model or a model with a combination of different snow properties and their interactions is the most parsimonious and best explains variance in Dall's sheep productivity. Sequential regression is done by including what is judged as the most important variable and then adding the residuals of the regression of a less important variable against the more important one. These residuals represent the effect of the second variable independent of the first. This process can be extrapolated to multiple collinear variables. See Graham 2003 (Confronting multicollinearity in ecological multiple regression; **https://doi.org/10.1890/02-3114**) and Dormann et al. 2013 (Collinearity: a review of methods to deal with it and a simulation study evaluating their performance; **https://doi.org/10.1111/j.1600-0587.2012.07348.x**) for further explanations of the method.

Results

Lines 389-394: The table or the figure should go in an appendix because they are mostly a repetition of the same information. I personally think the figure should stay.

Although the selected model was that of autumn snow depth, I think it would be interesting to have a figure showing the effect of the other more important snow properties. A 3 or 4 panel plot would be great.

Discussion

Line 442: Remove “an iconic mountain ungulate”.

Lines 442-444: “…conditions that inhibit Dall’s sheep, …” Dall sheep by themselves can’t be inhibited. Their productivity or recruitment, however, can. I think what is meant here is simply: “Snow conditions, most notably increased snow depth in the fall, were strongly associated with declines in Dall’s sheep productivity”.

Lines 457-461: Distinguishing between effects of predation and foraging efficiency/forage quantity seems far stretched given the results in this manuscript. Spring snow conditions can also influence productivity because of their influence on the length of the growing season. However, I agree that snow conditions in fall appear more important than snow conditions in spring for Dall’s sheep productivity.

Line 460: Remove “that”.

Line 470: I am not sure I understand this. Shouldn’t it be “…immediately after lambing.”.

Lines 486-492: Care must be taken when interpreting an AICc model selection. The fact that Fall forageable area was the third highest ranked model is not indicative that the “…methodology had some power in mapping the extent of snow conditions that could potentially impact foraging.”. AICc values are in no way representative of power nor do they represent the proportion of the variance explained. For instance, a model could be the highest ranked model from a candidate set based on an AICc model comparison and still explain <1% of the variance if the other models

are even worst. Instead, to evaluate and compare model performance, I suggest using the rsquared function from the piecewiseSEM package in R (https://rdrr.io/cran/piecewiseSEM/man/rsquared.html). This function returns (pseudo)-R^2 values for all generalized linear mixed effects models.
