# Supplementary material for: Seasonal influence of snow conditions on Dall’s sheep productivity in Wrangell-St Elias National Park and Preserve
Source: PLoS One. 2021 Feb 9;16(2):e0244787. doi: 10.1371/journal.pone.0244787 (PMC7872280; doi:10.1371/journal.pone.0244787)
Supplement: Attachment — Submitted filename: Response to Reviewers.docx [file pone.0244787.s022.docx]

Ottsjövägen 22

Årsta 12058

Sweden

16/12/2020

**Response to the review PONE-D-20-03620 ‘Seasonal influence of snow conditions on Dall’s sheep productivity in Wrangell-St Elias National Park and Preserve’**

Dear Dr Serrano,

We are grateful to you and the reviewers for the further comments on our manuscript and are glad that our original revisions were well received. As the comments were relatively few, I will address them individually in the following paragraphs.

In your emailed response you noted that Reviewer 3 was concerned with the R-squared values we report relative to other cited studies. To address this, I have included a comparison on lines 499 to 502 of our top-model to the R-squared reported by Rattenbury et al. (2018) for a region that is within our study area, Nabesna. We report an adjusted R-squared of 0.41, whereas Rattenbury et al. (2018), using the remote-sensing derived date of the end of the continuous snow season as their predictor of lamb-to-ewe ratios, report an R-squared of 0.33 (see figure 4 in Rattenbury et al.). The similar study of van de Kerk et al. (2018) only reported AICc for their various models, hence direct comparison is not possible. Of note is that Rattenbury et al. (2018) report a combined R-squared of 0.31 for all five Dall’s sheep count areas included in their study, and an R-squared of 0.65 for the Itkillik sub-area alone, which is the only sub-area to have an R-squared > 0.41. However, I believe it’s not appropriate to compare results from quite different Dall’s sheep ranges, Itkillik, for example, is in the Brooks Range where the snow season and landscape is dissimilar to the Wrangells, so only include Rattenbury et al.’s (2018) Nabesna result in the text. In response to why our R-squared could be perhaps thought of as relatively low, we offer the existing discussion between lines 539 and 552, which discusses other factors affecting Dall’s sheep productivity.

Reviewer 1 asked for the preliminary analysis of density dependence and delayed effects to be mentioned within the text. We appreciate this point and I have cleared up the discussion of this on lines 521 to 527 to be more explicit as to what we did to address in terms of density dependence. Likewise, I have hopefully clarified our delayed effect results (see line 407) and guide the reviewer to consider lines 534 to 538, where this result is reflected upon in the Discussion section. A further comment considered our choice of using ‘lamb-to-ewe’ to describe our metric of productivity and suggested using different terminology. We are sympathetic to the reviewer’s point, especially in respect to the potential inclusion of young-rams into the ‘ewe’ pool. However, we do feel confident that our explanation of the metric, and how it was derived, is clear with careful reading (see lines 192 to 204) and that it is appropriately and consistently termed in respect to similar, contemporary studies, e.g. van de Kerk et al. (2018) and Rattenbury et al. (2018). Reviewer 1 also makes a salient point on our hypotheses, questioning why we also analyse winter snow and climate conditions when our hypotheses only address fall and spring. This was an oversight on our part, so we have updated hypothesis 1 (lines 120 to 123) to now include; ‘…in which case snow conditions established in the fall months *and persisting through the winter months* should better explain summer lamb-to-ewe ratios’. The original idea in including the winter months in the analysis was to capture persistent effects of snow cover in the instance of a relatively mild and low-snow early fall followed by a relatively ‘snowy’ and cold late-fall and winter, which might still show a persistent effect on productivity vs spring conditions alone.

Reviewer 3 additionally discussed the location within the text of the results from the SnowModel calibration and the reporting of the bulk density RMSE in them. Prior to submission we considered whether the SnowModel calibration results should be contained within the Methods section but came to the opinion that, as they were results in and of themselves, they were more appropriately placed in the Results section. We also felt that it was possibly more readable in this format. The bulk density RMSE is now reported on line 382. Lastly, Reviewer 3 mentioned some typographical errors – after further proofreading we hope that we have caught these, especially those relating to incorrectly numbered references due to inadequate use of my referencing software.

We hope that you find the above and the updated manuscript to your satisfaction, and thank you again for the time taken to review it with care. We look forward to your response.

Sincerely,

Chris Cosgrove
